# Supplementary material for: Mechanical Properties of Flexible TPU-Based 3D Printed Lattice Structures: Role of Lattice Cut Direction and Architecture
Source: Polymers (Basel). 2021 Sep 3;13(17):2986. doi: 10.3390/polym13172986 (PMC8433625; doi:10.3390/polym13172986)
Supplement: Supplementary file 1 [file polymers-13-02986-s001.zip › polymers-1334042 -supply.pdf]

Supplementary materials

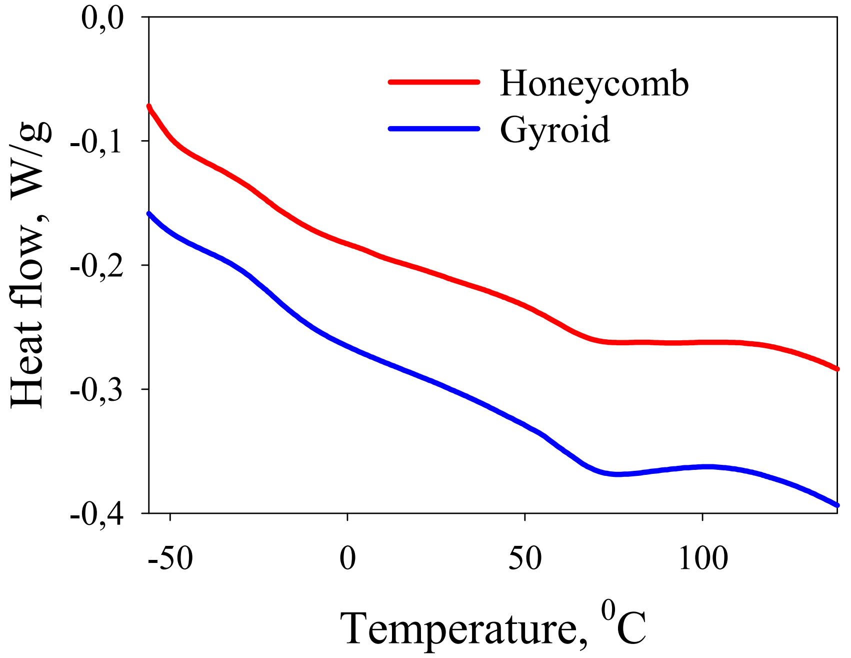

Figure 1S. DSC curves of lattice flexible TPU with honeycomb and gyroid architecture.

The recovery degree of lattice flexible TPU after compression tests was determined by the formula: $R_r = \left(\frac{h_{current}}{h_{initial}}\right) \times 100\%$, where $h_{current}$ is the current value of the sample height after unloading, h_{inital} is the initial value of the sample height before loading.

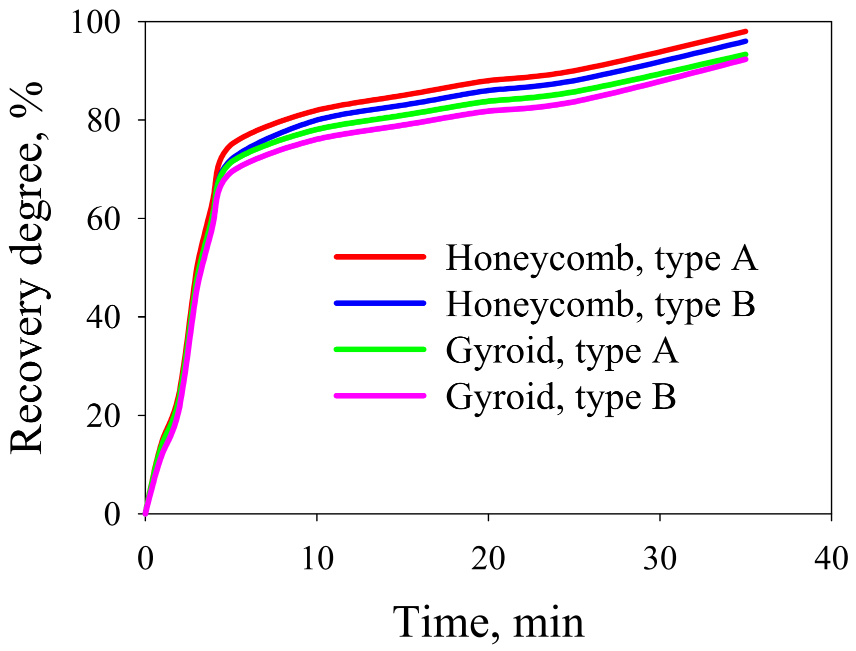

Figure 2S. The recovery degree of lattice flexible TPU with honeycomb and gyroid architecture over time after the compression tests.
